# Microbiome Composition and Function Drives Wound-Healing Impairment in the Female Genital Tract

**DOI:** 10.1371/journal.ppat.1005889

**Published:** 2016-09-22

**Authors:** Alexander S. Zevin, Irene Y. Xie, Kenzie Birse, Kelly Arnold, Laura Romas, Garrett Westmacott, Richard M. Novak, Stuart McCorrister, Lyle R. McKinnon, Craig R. Cohen, Romel Mackelprang, Jairam Lingappa, Doug A. Lauffenburger, Nichole R. Klatt, Adam D. Burgener

**Affiliations:** 1 Department of Pharmaceutics, Washington National Primate Research Center, University of Washington, Seattle, Washington, United States of America; 2 National HIV and Retrovirology Labs, JC Wilt Center for Infectious Diseases, Public Health Agency of Canada, Winnipeg, Canada; 3 Department of Medical Microbiology and Infectious Diseases, University of Manitoba, Winnipeg, Canada; 4 Department of Biomedical Engineering, University of Michigan, Ann Arbor, Michigan, United States of America; 5 Mass Spectrometry and Proteomics Core, National Microbiology Lab, Public Health Agency of Canada, Winnipeg, Canada; 6 Department of Medicine, University of Illinois at Chicago, Chicago, Illinois, United States of America; 7 Department of Obstetrics, Gynecology & Reproductive Sciences, University of California San Francisco, San Francisco, California, United States of America; 8 Department of Global Health, University of Washington, Seattle, Washington, United States of America; 9 Department of Medicine, University of Washington, Seattle, Washington, United States of America; 10 Department of Pediatrics, University of Washington, Seattle, Washington, United States of America; 11 Department of Biological Engineering, Massachusetts Institute of Technology, Cambridge, Massachusetts, United States of America; 12 Unit of Infectious Diseases, Department of Medicine, Center for Molecular Medicine, Karolinska Institute, Karolinska University Hospital, Stockholm, Sweden; Emory University, UNITED STATES

## Abstract

The mechanism(s) by which bacterial communities impact susceptibility to infectious diseases, such as HIV, and maintain female genital tract (FGT) health are poorly understood. Evaluation of FGT bacteria has predominantly been limited to studies of species abundance, but not bacterial function. We therefore sought to examine the relationship of bacterial community composition and function with mucosal epithelial barrier health in the context of bacterial vaginosis (BV) using metaproteomic, metagenomic, and *in vitro* approaches. We found highly diverse bacterial communities dominated by *Gardnerella vaginalis* associated with host epithelial barrier disruption and enhanced immune activation, and low diversity communities dominated by *Lactobacillus* species that associated with lower Nugent scores, reduced pH, and expression of host mucosal proteins important for maintaining epithelial integrity. Importantly, proteomic signatures of disrupted epithelial integrity associated with *G*. *vaginalis*-dominated communities in the absence of clinical BV diagnosis. Because traditional clinical assessments did not capture this, it likely represents a larger underrepresented phenomenon in populations with high prevalence of *G*. *vaginalis*. We finally demonstrated that soluble products derived from *G*. *vaginalis* inhibited wound healing, while those derived from *L*. *iners* did not, providing insight into functional mechanisms by which FGT bacterial communities affect epithelial barrier integrity.

## Author Summary

The female genital tract (FGT) is a key mucosal surface in the context of HIV transmission. *Lactobacillus* species are beneficial to the FGT, while *Garderella vaginalis* and other anaerobic bacteria are detrimental. Bacterial vaginosis (BV) is an inflammatory condition characterized by an outgrowth of *G*. *vaginalis* and other anaerobes, which is linked to increased HIV acquisition rates. However, the mechanism behind this remains unknown. Here, we used a novel proteomic approach to simultaneously evaluate host and bacterial functions in the FGT. We found that women with *G*. *vaginalis-*dominated FGT bacterial communities always displayed markers of decreased epithelial barrier integrity, and decreased wound healing capacity. We also demonstrated that the abundance of proteins from *G*. *vaginalis* associated with these signatures of disrupted epithelial integrity. Finally, we showed that products derived from *G*. *vaginalis* prevented healing of wounded cell monolayers while products derived from *L*. *iners* maintained the ability of the cell monolayers to close wounds. This study provides novel mechanistic insight into the link between BV and increased HIV acquisition rates.

## Introduction

Mucosal surfaces exposed to the external environment contain distinct bacterial communities that exist in relationship with the host and can contribute to health and functioning. These bacterial communities have been linked to several human diseases and overall health [1], and can vary between individuals, but also over time within the same person [2]. In the female genital tract (FGT), colonization by *Lactobacillus* species and other lactate-producing bacteria helps to inhibit colonization by pathogenic bacteria [3]. However, colonization by more diverse communities of anaerobic bacteria, notably *Gardnerella vaginalis*, is common [4], and often associated with the development of bacterial vaginosis (BV) [5]. BV is highly prevalent, affecting 4–58% of women globally; some areas, such as sub-Saharan Africa have rates as high as 55% [6]. BV is associated with significant health consequences, including pre-term birth, post-partum endometriosis, pelvic inflammatory disease, upper reproductive tract infections, and increased susceptibility to sexually transmitted infections (STI’s) [7, 8], with HIV being highly significant [9, 10]. Indeed, a recent meta-analysis linked BV to a 60% increase in HIV acquisition rates [11]. However, while these relationships between microbial composition and vaginal health have been described epidemiologically, there is limited understanding about the mechanisms underlying the impact of bacterial dysbiosis on the vaginal mucosa.

Maintenance of the mucosal barrier is critical for preventing invading microorganisms, including HIV, from penetrating into tissues and entering circulation [12]. Bacterial diversity in the FGT has been strongly associated with negative consequences for FGT mucosa. Highly diverse communities dominated by *G*. *vaginalis* and *Prevotella* are associated with upregulated expression of Toll-like Receptor (TLR) and NFkB pathways, leading to increased pro-inflammatory cytokine concentrations and activation of immune cells [13]. While it is widely appreciated that BV is associated with inflammation, the mechanism that elicits this inflammation or the bacterial proteins associated with inflammation remain unresolved [14], which may partly explain the limited effectiveness of antimicrobial treatment for BV [15–17]. Bacterial metabolites including hydrogen peroxide, antimicrobial peptides, and acids that reduce the FGT pH have been proposed to have an important impact in sustaining mucosal health [3]. Furthermore, the integrity of mucosal epithelial surfaces has been shown to depend on bacterial community composition in other diseases [18], and has been proposed to be important in the FGT during bacterial dysbiosis [19], but this has not been extensively studied. Each of these factors likely impact disease susceptibility independently, and a *Lactobacillus*-dominant microbiota likely contributes to many of these factors to maintain the function of the healthy FGT and inhibit infections. Taken together these studies suggest that host-microbe interactions are key to understanding negative consequences on vaginal health, yet this interaction remains poorly defined in human cohorts [20].

We sought to better understand the relationship between mucosal health and bacterial diversity using a combination of metaproteomics and metagenomics, which to our knowledge represents the first attempt at integrating these approaches to study the FGT. Indeed the functional diversity of the bacterial proteome, and how this relates to FGT health and inflammation has not been assessed comprehensively, and has largely been limited to 16S rRNA gene sequencing. Thus, we hypothesized that bacterial protein factors can influence FGT mucosal health and affect disease susceptibility. Here we characterized FGT bacterial communities in two distinct human cohorts, longitudinally and cross-sectionally, in asymptomatic and symptomatic women with BV, uncovering bacterial-host interactions leading to wound healing impairment.

## Results

### Vaginal bacterial proteome structure and diversity associate with dysbiosis and clinical BV

**Fig 1:**
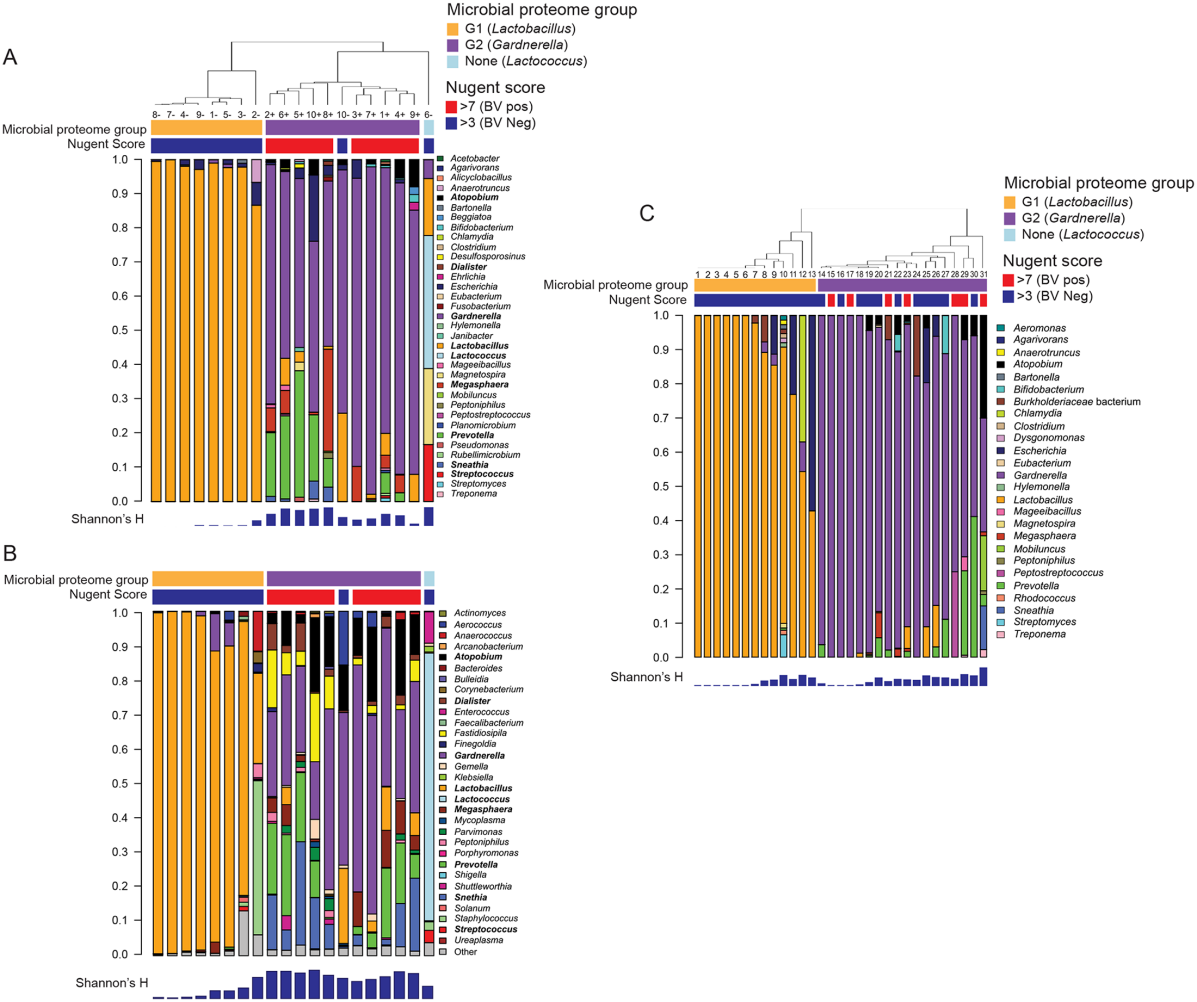
Mass spectrometry and 16S sequence analysis of cervicovaginal secretions show two distinct bacterial groups dominated by *Lactobacillus* (G1) or *Gardnerella* (G2) in two distinct cohorts. (A) Bacterial genera distributions detected in Cohort 1 using MS. Bacterial abundances from the MS data were calculated by summing normalized total spectral counts for all proteins associated with a genus/species. Clustering of samples was performed using unsupervised hierarchical linkage with average Euclidean distances of the proportional bacterial abundance in each sample. (B) Bacterial genera distributions for Cohort 1 detected using 16S rRNA gene sequencing shows close concordance to MS, and clustered into two groups based on *Lactobacillus* (G1) or *Gardnerella* (G2) dominance. Bacterial genera detected in Cohort 2 using MS (C) showed similar grouping as Cohort 1. Nugent scores (top) and bacterial alpha diversity (Shannon’s H index, bottom) are also shown.

Cervicovaginal secretion samples from two cohorts of women were evaluated to understand the mucosal environment associated with bacterial dysbiosis. We first assessed mucosal changes in women at BV+ or BV- time points (Cohort 1, n = 10), through a combination of mass spectrometry (MS) and 16S rRNA gene sequencing. MS analysis identified 1123 unique proteins, including 434 human and 689 bacterial proteins from 64 species. To assess the diversity of the bacterial proteome, we quantified the relative proteome load of each bacterial genus in each sample by summing the total number of protein spectral counts assigned to each genus, an approach previously shown to directly correlate with colony-forming units [21]. We clustered the bacterial proteomes from the twenty samples using unsupervised hierarchical clustering. Two major bacterial proteomes were identified, dominated by either *Lactobacillus iners* (Group 1, or G1) or *Gardnerella vaginalis* (Group 2, or G2) (Fig 1A, **species-level taxonomy shown in** S1A Fig), which were used for downstream comparisons. In G1, *L*. *iners* proteins accounted for 87–100% of the total protein load while in G2, *G*. *vaginalis* proteins accounted for 48–96%. Compared to those in G1, the bacterial proteomes in G2 displayed significantly higher species diversity (S1C Fig). G2 profiles also had higher overall bacterial protein load when normalized to the total protein content (0.34 log_10_, +2.2 fold higher; S1E Fig). *L*. *iners* dominated the FGT bacterial proteome of eight of the 10 patients from Cohort 1 at the time point without clinically diagnosed BV, but not the remaining two. Patient “10“, at the time point without BV, displayed a high abundance of *G*. *vaginalis* with a lower abundance of *L*. *iners*, and Patient “6”at the time point without BV had high levels of *Lactococcus lactis* and *Streptococcus mitis*. In contrast, all samples taken during episodes of BV had high abundances of proteins from *G*. *vaginalis*, *Prevotella* spp., *Streptococcus mitis*, *Escherichia coli*, and *Atopobium vaginae*, which have been previously identified as part of BV-associated bacterial communities [7].

Bacterial community composition for G1 and G2 was confirmed by 16S rRNA gene sequencing (Fig 1B). According to 16S rRNA gene sequencing, G1 communities were dominated by *Lactobacillus* spp. (26–99% of the total community), and G2 communities were dominated by *Gardnerella*, but at lower proportions than were detected by MS (17–66% of the total community). As with MS, bacterial genera detected in BV-positive individuals by 16S rRNA gene sequencing included *Sneathia*, *Prevotella*, *Atopobium*, *Megasphera*, and others. 16S rRNA gene sequencing also detected greater bacterial diversity in the G2 samples compared to G1 (S1C Fig). Several species detected by 16S and not by MS included *Leptotrichia*, *Fastidiosipila*, *Shuttleworthia*, and *Aerococcus*. Overall, this demonstrates significant heterogeneity in the structure of FGT bacterial communities between clinically defined BV and asymptomatic time points, that *G*. *vaginalis* and other anaerobes associate with BV, and that specific species dominate the bacterial proteome landscape in mucosal secretions.

### Vaginal bacterial proteome variation evident in asymptomatic women from an independent cohort

Mucosal samples from a separate group of 31 women from North America (Cohort 2) were analyzed to further evaluate associations between FGT bacterial proteome diversity and BV. MS analysis of Cohort 2 samples showed similar trends to that of Cohort 1 (Fig 1C, **species-level taxonomy shown in** S1B Fig), including *Lactobacillus* spp.*-*dominant (G1) and *G*. *vaginalis*-dominant (G2) communities. A wider distribution of lactobacilli including *L*. *iners*, *L*. *crispatus*, and *L*. *jensenii* was observed in G1 in Cohort 2 than Cohort 1. Varying abundances of other BV-associated bacteria including *Prevotella* spp., *Atopobium vaginae*, *Mobiliuncus mulieris*, and *Sneathia* sp. were also observed. In Cohort 2, there was no difference in the species diversity of G1 compared to G2 (S1D Fig). While all 7 women with BV clustered into G2, 46% of participants demonstrated a *G*. *vaginalis*-dominated proteome despite a lack of clinical BV diagnosis, consistent with the observation that not all women have *Lactobacillus*-dominant FGT microflora despite low Nugent scores. Also similar to Cohort 1, G2 in Cohort 2 had higher overall microbial proteome burden than G1 (1.5-fold higher; S1F Fig), indicating further changes in bacterial community function. This agrees with other studies showing *Lactobacillus* dominance varies between 37–90% of women, with greater diversity and variation in African women [5, 13, 22].

### Vaginal metaproteome profiles are not associated with clinical variables

As bacterial diversity has been associated with other biological variables, such as concurrent STI’s [23] and hormonal contraceptive usage [10], we compared clinical characteristics between *Lactobacillus* and *Gardnerella*-dominant groups **(Cohort 1-** Table 1; **Cohort 2-** Table 2). With the exception of BV status, we found no differences between G1 and G2 with respect to age, contraceptive use, antimicrobial usage, last menstrual period, detectible STI’s, or sexual practices in either cohort. There were differences in Amsel’s criteria collected from Cohort 2 (S1 Table) between G1 and G2, where vaginal pH, clue and white blood cell presence was higher in women with G2 bacterial proteome profiles, in agreement with clinical BV status. Overall, there was no evidence to support that vaginal bacterial profiles were related to exogenous hormonal contraceptive use, the menstrual cycle, sexual behaviors, or concurrent STI’s in these cohorts.

**Table 1:** Clinical characteristics of participants in Cohort 1 (Kenya).

Variable	All sample time points	G1	G2	P value
Age (avg)	37.8	37.7	37.8	0.96
Nugent score positive	11/20	1	10	<0.0001
Antimicrobial usage (Metronidazole)	0%	0%	0%	1.00
Actively cycling (%)	100%	100%	100%	1.00
Days since LMP if cycling (median)	12.5 (+/- 12.6)	14.5 (+/- 10)	11.5 (+/-4.8)	0.14
Contraceptive use	NoHCU (6/10, 60%), AnyHCU (3/10, 30%), Switched CU (1/10, 10%)	NoHCU (6/9, 67%), AnyHCU (3/9, 33%)	NoHCU (5/9, 56%), AnyHCU (4/9, 44%)	0.50
Any other STI (proportion)	3/20 (15%)	1/10, 1 *Trichomonas*, (same person both timepoints)	2/10, 1 *Trichomonas*, 1 *Chlamydia*	0.50
Number of sex acts w/partner	8.5	8.5 +/- 6.4	10 +/- 7.1	0.49
# condom sex acts w/ partner (median)	2 +/- 3.8	2 +/- 3.7	2 +/- 4.1	1.00
# unprotected condomless sex acts with partner (median)	3.5 +/- 5.9	3 +/- 6.9	4 +/- 5.4	0.48

HCU: hormonal contraceptive user

**Table 2:** Clinical characteristics of participants in Cohort 2 (North America).

Variable	G1	G2	P value
Age (avg.)	26.6	32	0.15
Nugent Score positive (7–10)	0/13	7/18	0.01
Antibiotic use reported	0%	0%	1.00
Actively cycling (%)	100%	100%	1.00
Days since LMP if cycling (median)	13	18	0.12
Contraceptive use	0	0	1.00
Any other STI (proportion)	0	0	1.00
Douching	0	0	1.00
Sex w/in last 24 hrs (%)	1/13 (7.7%)	2/18 (10.5)%	0.62
Condom usage (%)	Sometimes (6/13, 46%), Always (1/13, 7.7%), Blank (5/13, 38%)	Sometimes (16/18, 89%), Always (1/18, 5.6%), Blank (1/18, 5.6%)	0.46
Ethnicity	Black/non-hispanic (9), Asian (1), Hispanic (0), Caucasian (3)	Black/non-hispanic (15), Asian (1), Hispanic (1), Caucasian (0)	0.02

### *Lactobacillus* and *Gardnerella*-dominated FGT bacterial communities exhibit divergent functional traits

**Fig 2:**
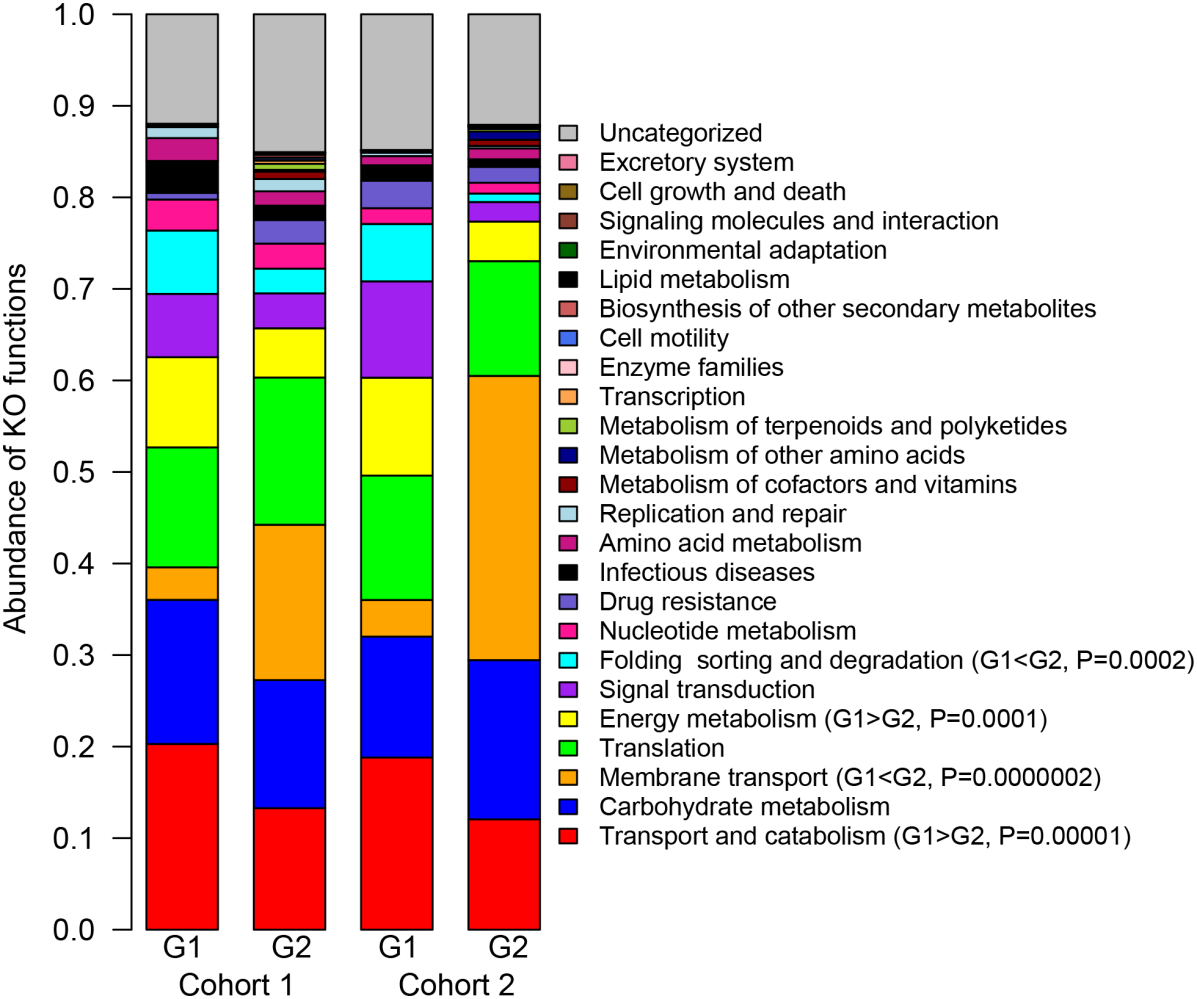
Cervicovaginal bacterial profiles display diverging functional traits. Bacterial proteome functions for both cohorts were determined by KEGG ontology. For both cohorts, G1 profiles (*Lactobacillus* dominant) showed increased abundance of proteins related to Transport and Catabolism and Energy Metabolism while G2 (*Gardnerella* dominant) showed increased abundances of proteins associated with Membrane Transport. Student’s *t*-tests were used to compare the abundances of each category between G1 and G2.

As the functional diversity of FGT-resident bacteria remained undefined we characterized the major bacterial pathways present in G1 and G2 profiles (Fig 2). Major functional categories represented in either group included transport and catabolism (G1 average 19.5%; G2 average 12.6%), carbohydrate metabolism (1 average 14.5%; G2 average 15.7%), as well as nucleotide and amino acid metabolism (G1 average 2.6%/1.8%; G2 average 2.0%/1.4%).

However, unique functional signatures were observed between G1 and G2 FGT bacterial communities. Across both cohorts, the G1 group showed significant enrichment of proteins involved in transport and catabolism (6.9% higher), energy metabolism (5.6% higher), and folding, sorting, and degradation (4.9% higher), while G2 was highly significantly enriched in membrane transport functions (22% higher). Twelve bacterial proteins were significantly differentially abundant after multiple comparison correction between G1 and G2 in Cohort 1 (S2 Table). Proteins enriched in G1 mostly belonged to *L*. *iners* proteins and were involved in homolactic fermentation of carbohydrates including glyceraldehyde-3-phosphate dehydrogenase (GAP-DH), pyruvate kinase (PK), and lactate dehydrogenase (LDH). Proteins enriched in G2 were all *G*. *vaginalis* proteins and included a MalE-type ABC sugar transport system periplasmic component (MAL-E ABC) and an alpha-1,4 glucan phosphorylase, an enzyme that degrades starch and glycogen, suggesting that *G*. *vaginalis* directs its metabolism towards liberation and uptake of extracellular saccharides. Although many of these proteins were also differentially abundant between G1 and G2 in Cohort 2 they did not pass multiple comparison correction. Overall this shows that ‘core’ functional pathways necessary to host-associated bacterial life within the FGT include carbohydrate, amino acid, and translational machinery, while perturbations to membrane transport and carbohydrate catabolism are likely important for pathogenic states.

### Vaginal epithelial wounding and immune activation associates with bacterial communities despite absence of clinical symptoms

Bacterial dysbiosis impacts HIV acquisition risk [10, 11, 24], reproductive health [7], and mucosal cellular activation [13], but the effect on the FGT is not well defined. Our analysis revealed that 69/434 (15.8%, 15 passing 5% FDR) and 64/434 (14.7%, 19 passing 5% FDR) host proteins were significantly differentially abundant between G1 and G2 profiles in Cohort 1 and 2, respectively. For Cohort 2, comparison based on bacterial groups rather than Nugent score criteria yielded greater host proteome differences, statistically (9.2% vs. 15.8%, *P*<0.05), and in magnitude (5 vs. 6 Log_2_ Fold Change; S1G/S1H Fig), suggesting that bacterial community composition, rather than clinical BV criteria, more accurately classifies mucosal inflammation. This comparison was not possible for Cohort 1, as all G2 profiles had clinically defined BV. Hierarchical cluster analysis revealed that longitudinal changes from G1 to G2 profiles in Cohort 1 were clearly distinguishable by two major branches of host proteins (S2A/S2B Fig). Proteins more abundant in G1 (Branch 1) associated with epidermis development and the cornified envelope, whereas G2 (Branch 2) showed increased factors involved in cytoskeletal-binding, threonine proteases involved in proteasome activity, as well as vesicular components and the melanosome. Many of these included S100 proteins and innate immune factors, important for antimicrobial defense based on gene ontology (DMBT1, CADH1, S10A7, EFHD2, S10AB, S10A6, TGM3, K2C1 S10A2). Similarly, in Cohort 2, hierarchical cluster analysis showed that proteins more abundant in G1 (Branch 1) associated with epidermis development, structural molecular activity, and the cornified envelope, while proteins elevated in G2 (Branch 2) also included ectoderm development and differentiation, although were related to cytoskeletal activity (S2C/S2D Fig). Many of these are important for leukocyte-mediated immunity and wounding responses based on their gene ontology (A1AT, IC1, GELS, CO3, PEBP1, PRDX1, PRDX2, CO4A, ANXA8).

**Fig 3:**
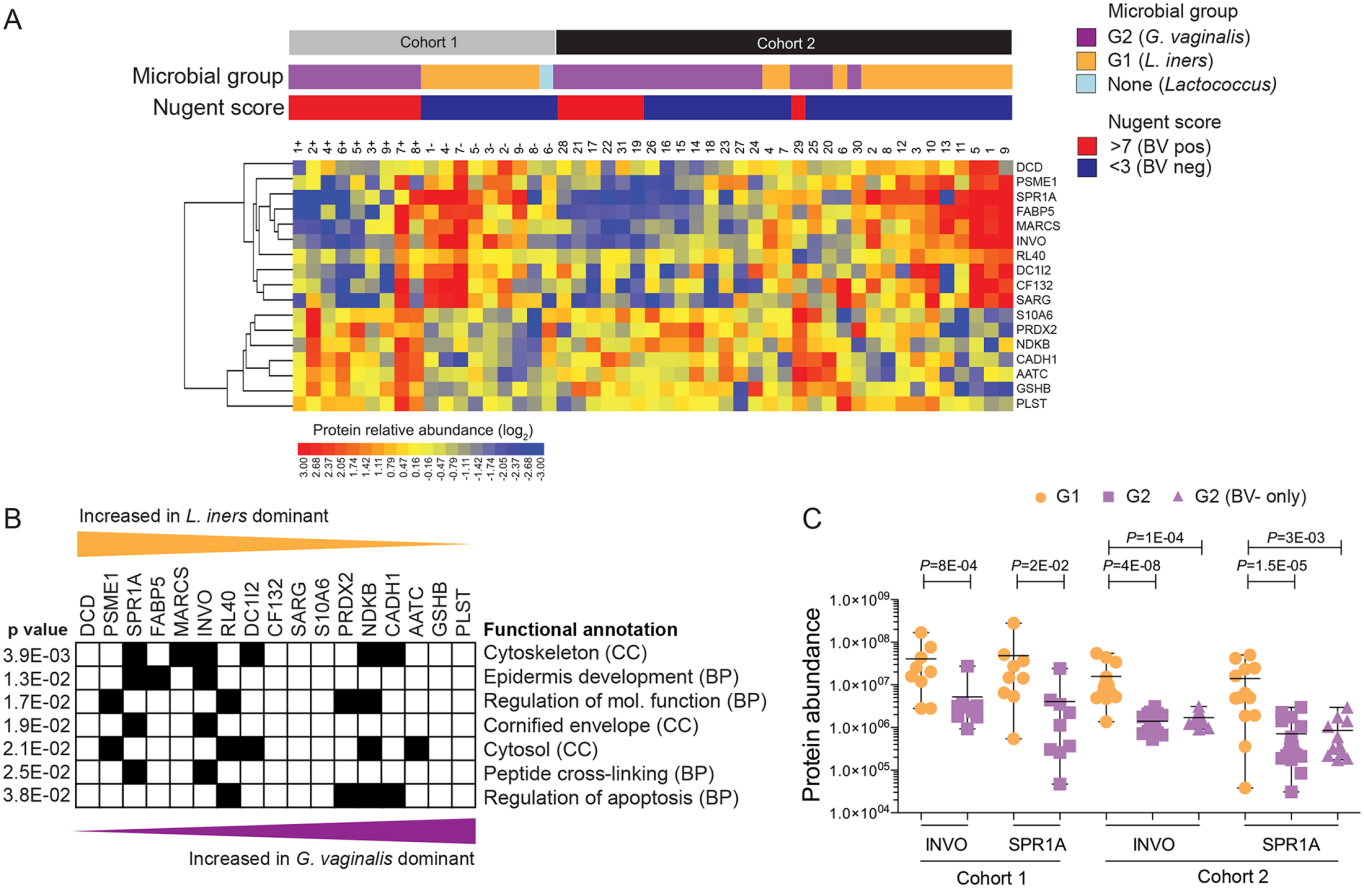
Vaginal epithelial barrier proteins vary significantly between microbial groups and are considerably lower in *Gardnerella* (G2) vs. *Lactobacillus* (G1) dominant profiles. **(A)** Heatmap of differentially abundant host proteins found in common between Cohort 1 (longitudinal) and Cohort 2 (cross-sectional) based on cervicovaginal microbiome profiles. **(B)** Functional annotation of differentially expressed proteins based on their gene ontology. Fisher’s exact test was used to compare annotated protein functions between G1 and G2 across both cohorts. **(C)** Lowered cornified envelope proteins (Involucrin and Small Proline-Rich Protein 1A) strongly correlate with *G*. *vaginalis* (G2) profiles, regardless of the presentation of clinical BV symptoms for both cohorts. Student’s *t*-test was used to compare the abundances of INVO and SPR1A between G1 and G2. BP: Biological process; CC: Cellular component.

Seventeen proteins were differentially abundant across both cohorts (Fig 3A). Host proteins more abundant in G2 profiles included apoptotic regulators (PRDX, NDKB, CADH1) and leukocyte migration factors (PLST), while G1 profiles showed increased keratinization, epidermis development, and cornified envelope (INVO, SPR1A) factors (Fig 3B). Of particular interest, the abundances of INVO and SPR1A were 14.7 and 7.2-fold lower in women G2 microbial profiles Cohorts 1 and 2, respectively. In Cohort 2, INVO and SPR1A were lower for women with G2 microbial profiles even if they had not been clinically diagnosed for BV (Fig 3C). These proteins are known to act as scaffolding for epidermal layers and are important for proper barrier function [25], and immunohistochemical analysis confirmed the presence of INVO and SPR1A in cervical and vaginal tissues, where they strongly associated with the squamous epithelium and stratum corneum in healthy FGT tissue (S3 Fig). Collectively these data show an association of heightened immune activation, apoptosis, and decreased epithelial barrier function in women with *G*. *vaginalis-*dominated bacterial profiles and that these effects are evident in *G*. *vaginalis*-dominated communities in the absence of clinical diagnosis.

### Functional variation in the vaginal microbiome correlates with epithelial wounding

**Fig 4:**
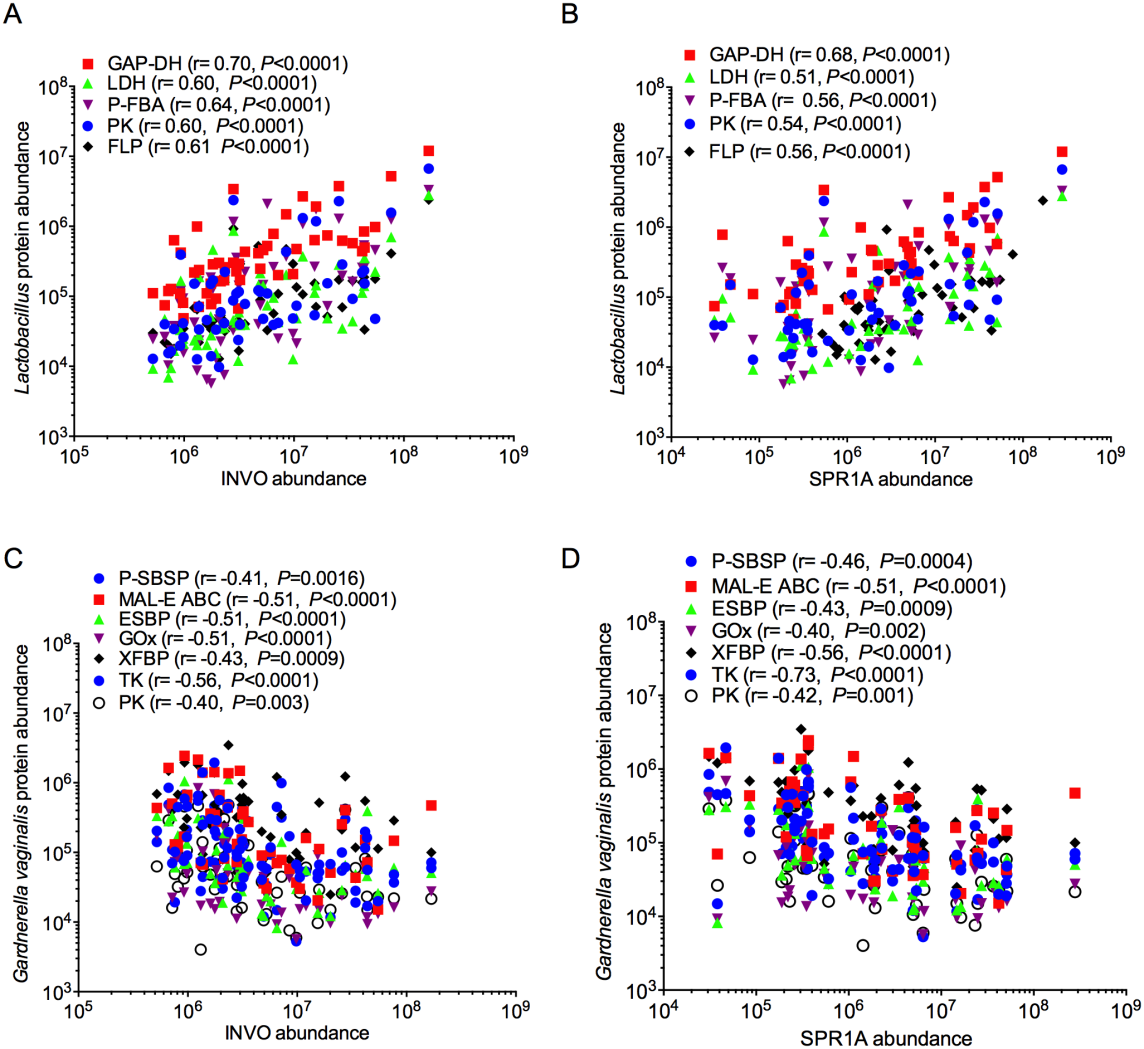
Bacterial proteins significantly correlate with vaginal epithelial barrier markers. Spearman correlations between **(A)** *Lactobacillus* proteins vs. INVO **(B)** *Lactobacillus* proteins vs. SPR1A; **(C)** *G*. *vaginalis* proteins vs. INVO; **(D)** *G*. *vaginalis* proteins vs. SPR1A. (SPR1A: Small proline-rich protein 1A; INVO, Involucrin) for both cohorts. Only proteins that passed multiple comparison correction testing in at least one comparison are shown (Bonferonni, *P* < 0.0003).

Due to the strong association of epithelial development pathways with different bacterial groups, we compared cornified envelope factors INVO and SPR1A to bacterial proteins. Nineteen bacterial proteins had strong associations in at least one comparison against either INVO or SPR1A after correcting for multiple comparisons. Proteins from *L*. *iners* that positively correlated with INVO and SPR1A were involved in Catabolism and Energy Metabolism pathways, including glycolysis and homolactic fermentation of sugars (Embden-Meyerhoff-Parnas (EMP) pathway) (Fig 4A/4B). These included a putative fructose 1,6-bisphosphate aldolase (PFBA), PK, GAP-DH, and LDH, as well as a ferritin-like protein (FLP), which is important for sequestering excess iron and preventing oxidative damage [26]. Bacterial proteins that negatively correlated with INVO and SPR1A belonged to alternate sugar metabolism pathways (phosphoketolase pathway), transport functions, and amino acid catabolism. The majority of these belonged to *G*. *vaginalis* (Fig 4C/4D), including D-xylulose 5-phosphate/D-fructose 6-phosphate phosphoketolase (XFBP), a putative sugar-binding secreted protein (P-SBSP), MAL-E ABC, an extracellular solute binding protein (ESBP), and glycine oxidase (GOx). A membrane protein from *Prevotella* sp., sharing sequence homology with SusD-like (starch-binding) protein, was also negatively associated (S4 Fig). Many associations with vaginal pH were also observed, including negative associations with enzymes from *L*. *iners* (PK, LDH, elongation factor tu, and FLP) (S5A Fig), and positive associations with enzymes from *G*. *vaginalis*, including GOx, which catalyzes the conversion of glycine into glyoxalate, ammonia, and hydrogen peroxide (S5B Fig). Therefore a clear relationship between metabolic function, epithelial barrier protein levels, and vaginal pH was observed, demonstrating that these microbial pathways may be an important component of mucosal barrier disruption and vaginal health.

### Soluble products derived from culture of *G*. *vaginalis* impair wound-healing capacity.

**Fig 5:**
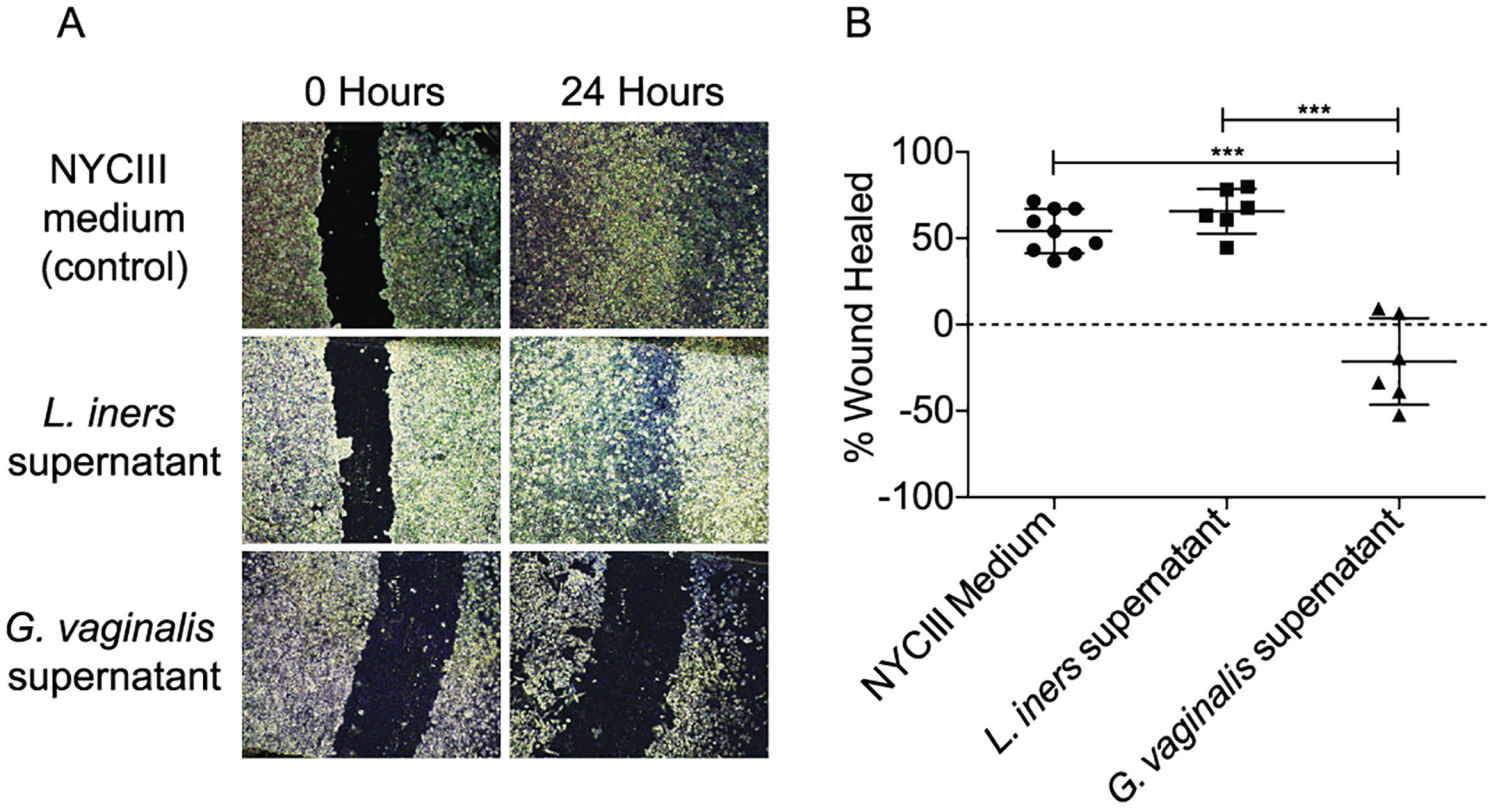
*G*. *vaginalis*-derived soluble products inhibit wound healing of cervical cell monolayers. (A) Representative light micrographs show that scratches in HeLa cell monolayers healed fully in the presence of NYCIII medium and *L*. *iners*, culture supernatants while HeLa cell monolayers exposed to *G*. *vaginalis* culture supernatants did not heal fully or expanded. (B) Statistical analysis of replicate co-culture experiments. Statistical significance was calculated using ANOVA with Bonferonni correction. ***P<0.001.

The association of bacterial communities with barrier integrity proteins led us to hypothesize that wound-healing capacity may be supported or inhibited by specific bacterial species and/or their products. We thus performed a classical wound-healing assay wherein we cultivated relevant cervical cell line (HeLa CCL-2) in the presence of supernatants derived from cultures of *L*. *iners* or *G*. *vaginalis*. Prior to adding culture supernatants, a wound was induced by scratching HeLa cell monolayers. Incubation of scratched monolayers with *L*. *iners* culture supernatant did not alter wound healing compared to the control incubations. However, incubation with *G*. *vaginalis* culture supernatants significantly reduced wound healing after 24 hours compared to both the control and *L*. *iners* conditions (Fig 5). These results confirm a relationship between soluble compounds produced by the major bacterial species of the G1 and G2 profiles and wound healing capacity. This implicates these species as important components or drivers of epithelial barrier repair, maintenance, and disruption in the FGT.

## Discussion

In this study, we demonstrated a novel metaproteomic approach to simultaneously assess bacterial diversity, abundance, and function, along with host barrier and inflammation processes, providing mechanistic insight relevant to women’s health. We described distinct vaginal bacterial proteome profiles that were dominated by *Lactobacillus* spp. (G1) or *G*. *vaginalis* (G2), where the latter associated with BV, increased community diversity, and significant divergence from normal metabolic function. We next demonstrated that bacterial functional profiles were significantly associated with cornified envelope factors in the FGT, and this was affected even in the absence of clinical diagnosis. Finally, we found that predominant species identified in this study, specifically *G*. *vaginalis* and *L*. *iners*, generate soluble products that disrupt or maintain the ability of cervical epithelial cells to repair and close wounds. Therefore, impaired wound healing is a potential mechanism by which key bacterial species may impact mucosal barrier function and therefore disease and/or HIV/STI infection risk. The association of vaginal inflammation and inflammatory vaginal bacteria with HIV susceptibility indicates that targeting this mechanism may lead to novel prevention strategies for HIV.

While increased diversity of bacterial communities has been linked to better mucosal functioning in the gut [27, 28], low-diversity bacterial communities are beneficial for the FGT [5], where increased diversity is strongly associated with BV [7]. Consistent with previous observations [5], many of the women in Cohort 2 with *G*. *vaginalis-*dominated communities were asymptomatic for BV (61%), further supporting the fact that Nugent score is underestimating the extent of non-*Lactobacillus* dominant communities. However, the effects on host epithelial pathways, including decreased integrity and increased inflammatory pathways were still evident in the absence of clinical diagnosis. *Lactobacillus* spp. and *G*. *vaginalis* proteins comprised more of the soluble proteome load than might be inferred from 16S rRNA gene sequencing, suggesting that these bacteria dominate the metabolic landscape of the FGT.

Metagenomic studies of the human microbiome have shown that core metabolic function is less variable than the community composition [2]. In agreement with this, we observed that the majority of assigned protein functions did not vary significantly, which likely represent core metabolic functions. However, some functions varied between G1 and G2, including increased carbohydrate metabolism, energy production, and folding/sorting functions in G1 to enhanced membrane transport and secretion of extracellular products in G2, with *L*. *iners* and *G*. *vaginalis* dominating these key functions. The increased abundances of enzymes important for sugar transport and starch and glycogen catabolism in G2 suggest that *G*. *vaginalis* may outcompete *Lactobacillus* spp. for the uptake of carbohydrate substrates. This agrees with a recent study showing that women with BV have significant metabolite alterations in cervicovaginal mucous, including lower levels of carbohydrates, amino acids, and lactate, accompanied by increased levels of amino acid catabolites and polyamines [29]. Overall, this demonstrates that increased bacterial diversity is associated with changes in key metabolic pathways, which allows for better understanding of dysbiosis in the FGT.

We found that G1 profiles from both cohorts strongly associated with cornified envelope factors, especially INVO and SPR1A, which are expressed in the upper layers of the vaginal and cervical epithelia, and aid in maintaining epithelial integrity. Our group has previously reported that increased levels of cervocovaginal CD4+ T cells associated with lower levels of cornified envelope factors [30], demonstrating the important link between vaginal epithelial integrity and HIV acquisition risk. G1 profiles were also associated with higher levels of antimicrobial peptides, such as dermcidin, which is important for host defense against microorganisms [31]. In comparison, the G2 bacterial profiles correlated with lower cornified envelope and epithelial barrier factors, increased cytoskeletal elements important for cell migration, and increased proteasome factors. This agrees with other studies showing that BV associates with activation of innate immune and inflammation pathways in the FGT, including increased complement [32], proteasome levels [33], and pro-inflammatory cytokines and activated CD4+ T-cells [13]. Importantly, G2 bacterial proteome profiles associated with decreased abundances of INVO and SPR1A regardless of clinical BV status. This finding demonstrates that current methods used to diagnose BV likely underestimate the true extent of bacterial dysbiosis on mucosal barrier function the FGT, as the Nugent Scores were poor predictors of BV, especially for Cohort 2. Thus, new methods to detect and treat *G*. *vaginalis* in the FGT could aid in reducing HIV acquisition risk by promoting mucosal and epithelial barrier integrity, and reduced inflammation.

Catabolic enzymes involved in homolactic fermentation of glucose from *Lactobacillus*, such as L-lactate dehydrogenase, correlated with higher epithelial barrier proteins, while membrane transporters, extracellular proteins, and alternate routes of carbohydrate metabolism (heterolactic fermentative or phosphoketolase pathways) from *G*. *vaginalis* were negatively correlated. In addition, GOx, was strongly correlated with increased vaginal pH, implicating a role of this enzyme in altered vaginal pH during dysbiosis. To our knowledge this is the first time these bacterial enzymes have been associated with epithelial disruption signatures and vaginal pH. Collectively, this shows a relationship between bacterial community structure, metabolic function, disruption of epithelial proteins important for barrier integrity, and overall vaginal health.

We also demonstrated that *G*. *vaginalis* culture supernatants inhibited healing of scratched HeLa cell monolayers while, *L*. *iners* culture supernatants maintained effective wound healing. Based on these data, *G*. *vaginalis* is likely an important component or a potential driver of subverting the wound healing process. While acknowledging that HeLa cell monolayers do not completely recapitulate the squamous epithelium or immune environment of the FGT, this nevertheless supports would healing as an underlying mechanism. Taken collectively, and considering the metaproteomic, metagenomic, and *in vitro* models, these data suggest that *G*. *vaginalis* releases a variety of extracellular products in the vaginal compartment that aid in uptake for nutrients, alter the vaginal microenvironment, contribute to innate immune activation, and prevent healing of the epithelial barrier. Future studies to identify exact protein pathways involved, how they may be altered, and more advance animal and engineered tissue models would help better decipher these host-microbiome interactions.

It is important to compare discuss the benefits and limitations of metaproteomics compared to 16S rRNA-based techniques to characterize microbial communities in the vaginal compartment. Both techniques are quantitative and spectral counts by MS have been shown to correlate directly to colony-forming units [21]. An advantage of 16S over MS is greater resolution of the overall community structure, and while we showed high sensitivity to identify species that were at 0.1% of the population by MS, 16S captured more overall bacterial species. It is likely that the larger dynamic range of the proteome over the genome is a large contributing factor to this observation. Both 16S and MS rely on curated databases to identify species and are subjected to this same limitation in availability and extensiveness of libraries. While databases for 16S rRNA genes are likely more comprehensive, proteomic libraries are growing and becoming more available. MS is advantageous in that it can provide direct species-level identification, which is not achievable through high-throughput 16S rRNA gene sequencing methods. Furthermore, metaproteomic analysis reveals bacterial functional and metabolic activity, which is not provided by 16S-based approaches. Prior studies have attempted to alleviate this using MS to correlate metabolite abundances with species abundances [34], through metagenomic studies [35], or by employing computational methods to estimate bacterial community functional capacity based on 16S rRNA gene signatures [36], but nevertheless represent indirect methods to evaluate bacterial community functionality. While 16S rRNA gene sequencing is a popular and well-validated method for studying microbial communities, the use of metaproteomic approaches provides complimentary and invaluable data on community structure, function, and host inflammation to better study host-bacterial relationships.

Our data provide novel mechanistic insight of how dysbiosis of vaginal bacterial communities may directly increase host susceptibility to infection through the disruption of epithelial barriers, inhibition of wound repair, and induction of inflammation. In the context of HIV transmission, inhibition of wound repair is under studied and may represent underlying mechanisms in other risk factors for HIV, including hormonal contraceptive usage, intravaginal practices, and other STI’s. These pathways may also impact the effectiveness or responsiveness to mucosa-targeted prevention technologies for other infections, such as microbicides or vaccines for HIV. In summary, this study delineated functional configurations of microbial communities that impact vaginal health during BV, providing new information on host-bacterial interactions, enabling future experiments to probe host-microbe relationships in the FGT that could have important implications for women’s health.

## Materials and Methods

### Ethics statement

All women who participated in this study provided written informed consent. The studies were approved by the University of Washington Human Subjects Review Committee, the Kenya Medical Research Institute (KEMRI), Human Subjects Committee of the University of Illinois at Chicago, and the Research Ethics Board of the University of Manitoba.

### Study cohorts

#### Study cohort 1 (Kenya)

This study included longitudinal samples and data collected from Kenyan female partners from HIV-1 serodiscordant couples enrolled in the Partners PrEP study[37]. HIV-1 seronegative partners were followed at monthly visits that included detailed assessments of behavioral and medical histories. For this study, we identified archived cervical swab samples from 10 women in the placebo arm of the Partners PrEP study collected at 2 time points: one at a visit where the participant tested positive for BV (Nugent’s score 7–10) and one at a visit where the participant tested negative for BV (Nugent’s score 0–3). Exclusion criteria included those who exhibited genital ulcer disease through self-report or on physical exam at the sample collection visit. All women remained HIV-1 negative throughout the study. Women were also evaluated at baseline for bacterial STIs, *Trichomonas vaginitis*, *Neisseria gonorrhoeae*, and *Chlamydia trachomatis* and treated if found to be positive.

#### Study cohort 2 (North America)

A total of 31 women were included from this study site from the University of Illinois as part of the UIC Project Wish cohort recruited for HIV prevention studies. Participants were between the ages of 18 to 47, were not on any form of hormonal contraception, and abstained from sexual intercourse, vaginal medication/creams, or douching for at least 24 hours prior to sample collection. Ethnicity was predominantly Black/non-Hispanic (n = 27), with few Caucasian (n = 3), Asian (n = 2), and Hispanic women (n = 1). All participants underwent testing for HIV, *Trichomonas vaginitis*, *Neisseria gonorrhoeae*, and *Chlamydia trachomatis* from vagina swabs taken at the time of collection and were excluded if positive.

### Cervicovaginal sample collection

#### Study cohort 1

Copan Floqswabs (Copan Diagnostics, Murrieta CA, USA) were used to collect vaginal samples and samples were stored frozen at -80°C until analysis. The median number of days between vaginal swab sample collection was 334 +/- 501 days.

#### Study cohort 2

Cervicovaginal lavage (CVL) samples were obtained by instilling 10 ml of saline solution over the surface of the vaginal vault and ectocervix. The saline lavage was then redrawn (8–10mL) using the same syringe with which it was instilled. All samples were immediately stored on wet ice and subsequently frozen at -80°C within 1 hour of sample collection.

#### Bacterial vaginosis criteria

Cervicovaginal samples were utilized for identification of BV using Nugent’s score criteria. Briefly, Gram-stained smears were assessed for absence of *Lactobacillus* (scored 0–4), presence of *Gardnerella* and *Bacteroides* morphotypes (0–4), and presence of curved gram-variable rods (0–2) for a total score of 0–10 wherein 0–3 is healthy, 4–6 is intermediate, and 7–10 is diagnostic for BV. For Cohort 2, Amsel criteria were also collected including vaginal pH, clue cells on wet mount, and positive whiff test, and incorporated for a positive classification for BV (any 3 of the 4 criteria).

### Sample preparation for mass spectrometry

Vaginal swabs were eluted with 2 x 250ul washes in PBS (pH 7.0). Swab eluates (Cohort 1) or CVL samples (Cohort 2) were then centrifuged in SpinX tubes with a bonded fritted bottom (Corning, Corning, NY), and protein content determined by BCA assay (Novagen, Bilerica, MA). Proteins were then denatured, reduced, alkylated, digested into peptides, and prepared for mass spectrometry as described previously [38]. Detailed methods for this process are available in S1 Methods.

### Mass spectrometry analysis

Briefly, peptide samples were injected into a nano-flow LC system (Easy nLC, Thermo Fisher) connected inline to a LTQ Orbitrap Velos (Thermo Fisher) mass spectrometer, and analyzed in a label-free manner as described previously [38]. Peptide identity searching was performed with Mascot v2.4.0 (Matrix Science) against a manually curated database comprised of the SwissProt Human & Bacteria (June 2015) and UniProtKB/Trembl All Bacteria databases (August 2015). A decoy database was included to determine the rate of false discovery. Protein identifications were confirmed using Scaffold (v 4.4.1, Proteome Software) with confidence thresholds set at 95% protein identification confidence, requiring at least 2 unique peptides and 80% peptide identification confidence. A combination of label-free methods was used for protein quantitation: spectral counting (for microbial proteins and bacterial diversity clustering, see below) and area-under-the-curve quantitation (Progenesis LC-MS software (v4.0, Nonlinear Dynamics)). Criteria for assigning presence of microbial proteins included those that had at least 1 peptide in one sample, and at least 2 peptides per protein across all samples. These parameters resulted in a false discovery rate below 3.1% based on the search results run against Mascot’s generated decoy database. For the latter, only proteins that had an average co-variance of <25% (575 proteins), as determined through measurements of standard reference sample run at 10 sample intervals (total 7 times), were utilized in downstream analysis to exclude proteins with higher technical measurement variability. Complete details of liquid chromatography and mass spectrometry instrument settings are as described previously [38].

### Proteomic data analysis

#### Human proteome

Protein relative abundance values were obtained by dividing by average intensity across all samples, followed by log transformation (base 2). Graphical representations of proteomic data were generated in R. Differentially abundant proteins were clustered using unsupervised average linkage and Pearson correlation coefficient as the distance metric. A complete list of host proteins detected in this study is available in S1 Data Set.

#### Microbial proteome

Microbial abundance was calculated by summing normalized total spectral counts for all proteins associated with a genus/species. Unsupervised hierarchical linkage with average Euclidean distances was performed in MATLAB using the proportional microbial abundance in each sample, and stacked bar charts generated in R. K number annotations were assigned using GhostKOALA to obtain KEGG category information^29^. KEGG ontology (KO) assignments were manually curated to remove 7 categories associated with organism-level functions and 1 general “Overview” category. All proteins associated with these 8 categories were associated with had at least one other category. The average number of KO categories with which each unique protein associated was 1.97 with a maximum of 7. Cumulative functional abundance for each category was calculated by summing abundances of all associated proteins, and proteins belonging to multiple categories contributed to each of those associated. A complete list of bacterial proteins detected in this study is available in S2 Data Set, and spectral counts for bacterial proteins are available in S3 Data Set.

#### DNA extraction and 16S rRNA gene sequencing

Total genomic DNA from eluted swab (Cohort 1) or CVL (Cohort 2) samples was extracted using the DNeasy Blood and Tissue Kit (Qiagen, Valencia, CA) with modifications to enhance lysis, as previously described [39]. The Microbiome Analysis Laboratory at Arizona State University performed V3–V4 region 16S rRNA gene sequencing using the Illumina MiSeq sequencing platform, and sequences were analyzed using the QIIME software package [40]. Complete details for DNA extraction and sequence analysis are available in S1 Methods. All sequence data has been deposited to the NCBI SRA under BioProject Accession Number PRJNA317390.

### Pathway and biofunctional analysis

Biological/molecular functions and cellular components were annotated based on gene ontologies using the DAVID Bioinformatics Resource (v6.7) [41], which calculates a modified Fisher’s Exact *P* value to determine the probability that the association between each protein in the dataset and functional pathway is random. Functional categories were considered to be those with *P*-values < 0.05 (Benjamini Hochberg adjusted) and at least 3 proteins selected to be positive associations.

### Cell lines, bacterial strains, and culture conditions

HeLa (ATCC CCL-2) cells were obtained as a gift from the laboratory of Dr. Shiu-Lok Hu (University of Washington), and were maintained in Dulbecco’s Modified Eagle’s Medium (DMEM) supplemented with 4.5 g/L glucose, L-glutamine, 10% (v/v) fetal bovine serum (Corning), and 1% (v/v) penicillin/streptomycin/amphotericin B solution (Gibco). HeLa cells were incubated at 37°C with air/5% CO_2_ atmosphere. *Gardnerella vaginalis* ATCC 14018 and *Lactobacillus iners* ATCC 55195 were obtained from the American Type Culture Collection, and were maintained using HBT-Bilayer medium (BD) and NYCIII liquid medium with incubation at 37°C with air/5% CO_2_ atmosphere. Frozen stocks were stored in 20% (v/v) glycerol at -80°C.

### Wound-healing assay

To assess the impact of different bacteria on the ability of cervical epithelial cells to repair wounds, we utilized the well-established *in vitro* scratch assay [42]. To prepare live bacteria and culture supernatants for the wound-healing assay, overnight cultures of *L*. *iners* and *G*. *vaginalis* in NYCIII medium were grown as described above. Wells of a 24-well tissue culture plate (Corning) were initially seeded with 50,000 HeLa cells in a volume of 500 μL DMEM and incubated at 37°C under 5% CO_2_ until a confluent cell monolayer had formed. Monolayers in each well were then scratched using a sterile P200 pipette tip. Live bacteria, bacterial culture supernatants, or control solutions were then added to the wells. Images at five reference points per well were captured using a Nikon Eclipse TS100 microscope equipped with a Nikon DS-Ri1 camera and the size of the scratch at each reference point was manually analyzed using the ImageJ software. The size of the wound was determined immediately after beginning (t = 0) the experiment and then again after 24 hours (t = 24) of incubation at 37°C with air/5% CO_2_ atmosphere. Additional information on wound-healing assays is available in S1 Methods.

#### Statistical analysis

Independent (Mann-Whitney U-test) or paired sample *t-*tests were used on normalized proteomic data for statistical comparisons. Unless otherwise noted, significant changes were defined as those with a *P*-value below 0.05 after adjusting to a local false discovery rate and multiple comparisons [43]. Chi-squared tests were used for categorical variables. For the wound healing assay, the difference between the size of the scratch at t = 0 and t = 24 at each reference point was calculated and normalized to the initial scratch size to determine the percent of the wound that had healed after 24 hours. ANOVA with Bonferroni correction was conducted in GraphPad Prism (GraphPad).

## Supporting Information

S1 Fig Vaginal bacterial species diversity, protein abundance, and relationship to host protein expression as detected by mass spectrometry. **(A)** Bacterial species and proteome diversity for Cohort 1. Most of the G1 communities were dominated by *L*. *iners*, while *G*. *vaginalis*, *P*. *amnii*, *Megasphaera* sp., and others dominated the G2 communities. **(B)** Bacterial species and proteome diversity for Cohort 2. G1 communities were dominated by either *L*. *iners*, *L*. *crispatus*, but also had the presence of *L*. *gasseri*, and *L*. *jensenii*. G2 communities were heavily dominated by *G*. *vaginalis*. **(C)** Shannon Diversity detected for Cohort 1. The G2 group always had higher diversity than the G1 group. **(D)** Shannon Diversity detected for Cohort 2. There was no difference in the diversity between the G1 and G2 groups. **(E/F)** Total bacterial proteome abudance differences between women with either a *G*. *vaginalis* or *L*. *iners* bacterial proteome profile for Cohort 1 **(E)** and Cohort 2 **(F)**. These graphs show bacterial protein levels (y axis) as a function of abundance rank (x axis) in decreasing order. Median levels are shown (log10), and student *t-*tests (Cohort 1: paired t test, parametric; Cohort 2: unpaired t test, parametric) were used to determine statistical differences. *G*. *vaginalis*-dominant profiles always had increased protein load compared to those dominated by *Lactobacillus*. (G/H) Volcano plots depicting host protein expression differences using clinical BV status **(G)** or bacterial community profile **(H)**. The Y-axis of the volcano plot denotes statistical significance, and the x-axis the fold-change (FC). Comparison of G1 and G2 community profiles, rather than BV criteria, yielded more statistically relevant differences between the host proteome.
(TIF)

S2 Fig Vaginal mucosal inflammation pathways of women from Cohort 1 and Cohort 2 associated with bacterial community composition. Heatmaps of differentially abundant host proteins (*P*<0.05) in cervicovaginal secretions of women based on vaginal bacterial communities. **(A)** Differentially abundant host proteins for Cohort 1; **(B)** Functional annotation of differentially expressed proteins based on their gene ontology for Cohort 1. **(C)** Heatmap of differentially abundant host proteins for Cohort 2; **(D)** Functional annotation of differentially expressed proteins based on their gene ontology for Cohort 2. BP: Biological process; CC: Cellular component; MF: Molecular function.
(TIF)

S3 Fig Immunohistochemical images of epithelial barrier proteins involucrin and small proline-rich protein 1A in the vagina and cervix. Immunohistochemical analysis of cervical and vaginal tissue revealed prominent expression of INVO and SPR1A in top layers of the squamous epithelium in cervix and vagina, with both showing association with the cytoplasm and nucleus membrane. Protein was not detected in glandular cells in cervix/endocervix.
(TIF)

S4 Fig Membrane protein from *Prevotella* sp. that significantly negatively correlated with the abundances of INVO (left) and SPR1A (right) for both cohorts. (TIFF)

S5 Fig Bacterial proteins that significantly correlate with pH in the cervicovaginal epithelium. Four *L*. *iners* proteins negatively correlated with pH, including lactate dehydrogenase (LDH) **(A)**, and three *G*. *vaginalis* proteins positively correlated with pH, including glycine oxidase (GOx) **(B)**.
(TIFF)

S1 Table Amsel criteria for Cohort 2 (North America). (DOCX)

S2 Table Proteins determined to be significantly differentially abundant between G1 and G2 in Cohort 1 (BH *P*: Bejamani-Hochberg corrected *P* value; *P*<0.001). (DOCX)

S1 Methods Containing information on mass spectrometry, DNA extraction and 16S rRNA gene sequencing, and wound-healing assay. (DOCX)

S1 Data Set Human proteins detected by mass spectrometry (are under curve integration (Progenesis)). (XLSX)

S2 Data Set Bacterial proteins detected by mass spectrometry (area under curve integration (Progenesis)). (XLSX)

S3 Data Set Bacterial proteins detected by mass spectrometry (spectral counting (Scaffold)). (XLSX)

## References

[1] Costello EK, Lauber CL, Hamady M, Fierer N, Gordon JI, Knight R. Bacterial community variation in human body habitats across space and time. Science. 2009;326(5960):1694–7. 10.1126/science.1177486 19892944 PMC3602444

[2] Human Microbiome Project C. Structure, function and diversity of the healthy human microbiome. Nature. 2012;486(7402):207–14. 10.1038/nature11234 22699609 PMC3564958

[3] Aroutcheva A, Gariti D, Simon M, Shott S, Faro J, Simoes JA, et al Defense factors of vaginal lactobacilli. Am J Obstet Gynecol. 2001;185(2):375–9. 11518895 10.1067/mob.2001.115867

[4] Fredricks DN, Fiedler TL, Marrazzo JM. Molecular identification of bacteria associated with bacterial vaginosis. N Engl J Med. 2005;353(18):1899–911. 16267321 10.1056/NEJMoa043802

[5] Ravel J, Gajer P, Abdo Z, Schneider GM, Koenig SSK, McCulle SL, et al Vaginal microbiome of reproductive-age women. Proc Natl Acad Sci. 2011;108(suppl. 1):4680–7. 10.1073/pnas.1002611107 20534435 PMC3063603

[6] Kenyon C, Colebunders R, Crucitti T. The global epidemiology of bacterial vaginosis: a systematic review. Am J Obstet Gynecol. 2013;209(6):505–23. 10.1016/j.ajog.2013.05.006 23659989

[7] Srinivasan S, Hoffman NG, Morgan MT, Matsen FA, Fiedler TL, Hall RW, et al Bacterial communities in women with bacterial vaginosis: high resolution phylogenetic analyses reveal relationships of microbiota to clinical criteria. PloS one. 2012;7(6):e37818 10.1371/journal.pone.0037818 22719852 PMC3377712

[8] Fethers KA, Fairley CK, Hocking JS, Gurrin LC, Bradshaw CS. Sexual risk factors and bacterial vaginosis: a systematic review and meta-analysis. Clin Infect Dis. 2008;47(11):1426–35. 10.1086/592974 18947329

[9] Shin LY, Kaul R. Stay it with flora: maintaining vaginal health as a possible avenue for prevention of human immunodeficiency virus acquisition. The Journal of infectious diseases. 2008;197(10):1355–7. 10.1086/587491 18444792

[10] Buvé A, Jespers V, Crucitti T, Fichorova RN. The vaginal microbiota and susceptibility to HIV. Aids. 2014;28(16):2333–44. 25389548 10.1097/qad.0000000000000432

[11] Atashili J, Poole C, Ndumbe PM, Adimora AA, Smith JS. Bacterial vaginosis and HIV acquisition: a meta-analysis of published studies. Aids. 2008;22(12):1493–501. 10.1097/QAD.0b013e3283021a37 18614873 PMC2788489

[12] Burgener A, McGowan I, Klatt NR. HIV and mucosal barrier interactions: consequences for transmission and pathogenesis. Current opinion in immunology. 2015;36:22–30. 10.1016/j.coi.2015.06.004 26151777

[13] Anahtar MN, Byrne EH, Doherty KE, Bowman BA, Yamamoto HS, Soumillon M, et al Cervicovaginal bacteria are a major modulator of host inflammatory responses in the female genital tract. Immunity. 2015;42(5):965–76. 10.1016/j.immuni.2015.04.019 25992865 PMC4461369

[14] Mitchell C, Marrazzo J. Bacterial vaginosis and the cervicovaginal immune response. Am J Reprod Immunol. 2014;71(6):555–63. 10.1111/aji.12264 24832618 PMC4128638

[15] McClelland RS, Balkus JE, Lee J, Anzala O, Kimani J, Schwebke J, et al Randomized Trial of Periodic Presumptive Treatment With High-Dose Intravaginal Metronidazole and Miconazole to Prevent Vaginal Infections in HIV-negative Women. The Journal of infectious diseases. 2015;211(12):1875–82. 10.1093/infdis/jiu818 25526757 PMC4836721

[16] McClelland RS, Richardson BA, Hassan WM, Chohan V, Lavreys L, Mandaliya K, et al Improvement of vaginal health for Kenyan women at risk for acquisition of human immunodeficiency virus type 1: results of a randomized trial. The Journal of infectious diseases. 2008;197(10):1361–8. 10.1086/587490 18444793 PMC4122228

[17] Wilson J. Managing recurrent bacterial vaginosis. Sexually Transmitted Infections. 2004;80(1):8–11. 14755028 10.1136/sti.2002.002733 PMC1758381

[18] Mathewson ND, Jenq R, Mathew AV, Koenigsknecht M, Hanash A, Toubai T, et al Gut microbiome-derived metabolites modulate intestinal epithelial cell damage and mitigate graft-versus-host disease. Nat Immunol. 2016;17(5):505–13. 10.1038/ni.3400 26998764 PMC4836986

[19] Thurman AR, Doncel GF. Innate immunity and inflammatory response to Trichomonas vaginalis and bacterial vaginosis: relationship to HIV acquisition. Am J Reprod Immunol. 2011;65(2):89–98. 10.1111/j.1600-0897.2010.00902.x 20678168

[20] Petrova MI, van den Broek M, Balzarini J, Vanderleyden J, Lebeer S. Vaginal microbiota and its role in HIV transmission and infection. FEMS microbiology reviews. 2013;37(5):762–92. 10.1111/1574-6976.12029 23789590

[21] Tanca A, Palomba A, Salvatore P, Deligios M, Fraumene C, Manghina V, et al A straightforward and efficient analytical pipeline for metaproteome characterization. Microbiome. 2014;2(49). 10.1186/s40168-014-0049-2 PMC4266899 25516796

[22] Zhou X, Brown CJ, Abdo Z, Davis CC, Hansmann MA, Joyce P, et al Differences in the composition of vaginal microbial communities found in healthy Caucasian and black women. The ISME journal. 2007;1(2):121–33. 18043622 10.1038/ismej.2007.12

[23] Brotman RM, Klebanoff MA, Nansel TR, Yu KF, Andrews WW, Zhang J, et al Bacterial vaginosis assessed by gram stain and diminished colonization resistance to incident gonococcal, chlamydial, and trichomonal genital infection. The Journal of infectious diseases. 2010;202(12):1907–15. 10.1086/657320 21067371 PMC3053135

[24] Cohen CR, Lingappa JR, Baeten JM, Ngayo MO, Spiegel CA, Hong T, et al Bacterial vaginosis associated with increased risk of female-to-male HIV-1 transmission: a prospective cohort analysis among African couples. PLoS Med. 2012;9(6):e1001251 10.1371/journal.pmed.1001251 22745608 PMC3383741

[25] Candi E, Schmidt R, Melino G. The cornified envelope: a model of cell death in the skin. Nature reviews Molecular cell biology. 2005;6(4):328–40. 15803139 10.1038/nrm1619

[26] Smith JL. The physiological role of ferritin-like compounds in bacteria. Crit Rev Microbiol. 2004;30(3):173–85. 15490969 10.1080/10408410490435151

[27] Turnbaugh PJ, Hamady M, Yatsunenko T, Cantarel BL, Duncan A, Ley RE, et al A core gut microbiome in obese and lean twins. Nature. 2009;457(7228):480–4. 10.1038/nature07540 19043404 PMC2677729

[28] Qin J, Li R, Raes J, Arumugam M, Burgdorf KS, Manichanh C, et al A human gut microbial gene catalogue established by metagenomic sequencing. Nature. 2010;464(7285):59–65. 10.1038/nature08821 20203603 PMC3779803

[29] Srinivasan S, Morgan MT, Fiedler TL, Djukovic D, Hoffman NG, Raftery D, et al Metabolic signatures of bacterial vaginosis. mBio. 2015;6(2). 10.1128/mBio.00204-15 PMC4453549 25873373

[30] Arnold KB, Burgener A, Birse K, Romas L, Dunphy LJ, Shahabi K, et al Increased levels of inflammatory cytokines in the female reproductive tract are associated with altered expression of proteases, mucosal barrier proteins, and an influx of HIV-susceptible target cells. Mucosal immunology. 2015;9(1):194–205. 10.1038/mi.2015.51 26104913

[31] Burian M, Schittek B. The secrets of dermcidin action. Int J Med Microbiol. 2015;305(2):283–6. 10.1016/j.ijmm.2014.12.012 25596890

[32] Cruciani F, Wasinger V, Turroni S, Calanni F, Donders G, Brigidi P, et al Proteome profiles of vaginal fluids from women affected by bacterial vaginosis and healthy controls: outcomes of rifaximin treatment. J Antimicrob Chemother. 2013;68(11):2648–59. 10.1093/jac/dkt244 23798671

[33] Borgdorff H, Gautam R, Armstrong SD, Xia D, Ndayisaba GF, van Teijlingen NH, et al Cervicovaginal microbiome dysbiosis is associated with proteome changes related to alterations of the cervicovaginal mucosal barrier. Mucosal immunology. 2015;9(3):621–33. 10.1038/mi.2015.86 26349657

[34] Srinivasan S, Morgan MT, Fiedler TL, Djukovic D, Hoffman NG, Raftery D, et al Metabolic Signatures of Bacterial Vaginosis. mBio. 2015;6(2):e00204–15. 10.1128/mBio.00204-15 25873373 PMC4453549

[35] Gill SR, Pop M, DeBoy RT, Eckburg PB, Turnbaugh PJ, Samuel BS, et al Metagenomic Analysis of the Human Distal Gut Microbiome. Science. 2006;312(5778):1355–59. 16741115 10.1126/science.1124234 PMC3027896

[36] Langille MG, Zaneveld J, Caporaso JG, McDonald D, Knights D, Reyes JA, et al Predictive functional profiling of microbial communities using 16S rRNA marker gene sequences. Nature biotechnology. 2013;31(9):814–21. 10.1038/nbt.2676 23975157 PMC3819121

[37] Baeten JM, Donnell D, Ndase P, Mugo NR, Campbell JD, Wangisi J, et al Antiretroviral prophylaxis for HIV prevention in heterosexual men and women. N Engl J Med. 2012;367(5):399–410. 10.1056/NEJMoa1108524 22784037 PMC3770474

[38] Birse K, Arnold KB, Novak RM, McCorrister S, Shaw S, Westmacott GR, et al Molecular signatures of immune activation and epithelial barrier remodeling are enhanced during the luteal phase of the menstrual cycle: implications for HIV susceptibility. Journal of virology. 2015;89(17):8793–805. 10.1128/JVI.00756-15 26085144 PMC4524071

[39] Zevin AS, Rittmann BE, Krajmalnik-Brown R. The source of inoculum drives bacterial community structure in Synechocystis sp. PCC6803-based photobioreactors. Algal Research. 2016;13:109–15.

[40] Caporaso JG, Kuczynski J, Stombaugh J, Bittinger K, Bushman FD, Costello EK, et al QIIME allows analysis of high- throughput community sequencing data. Nature methods. 2010;7(5):335–6. 10.1038/nmeth.f.303 20383131 PMC3156573

[41] Huang da W, Sherman BT, Lempicki RA. Systematic and integrative analysis of large gene lists using DAVID bioinformatics resources. Nature protocols. 2009;4(1):44–57. 10.1038/nprot.2008.211 19131956

[42] Liang CC, Park AY, Guan JL. In vitro scratch assay: a convenient and inexpensive method for analysis of cell migration in vitro. Nature protocols. 2007;2(2):329–33. 17406593 10.1038/nprot.2007.30

[43] Benjamini Y, Hochberg Y. Controlling the False Discovery Rate- a Practical and Powerful Approach to Multiple Testing. Journal of the Royal Statistical Society. 1995;57(1):289–300.

