# Supplementary figures and images for: Microbiome Composition and Function Drives Wound-Healing Impairment in the Female Genital Tract
Source: PLoS Pathog. 2016 Sep 22;12(9):e1005889. doi: 10.1371/journal.ppat.1005889 (PMC5033340; doi:10.1371/journal.ppat.1005889)

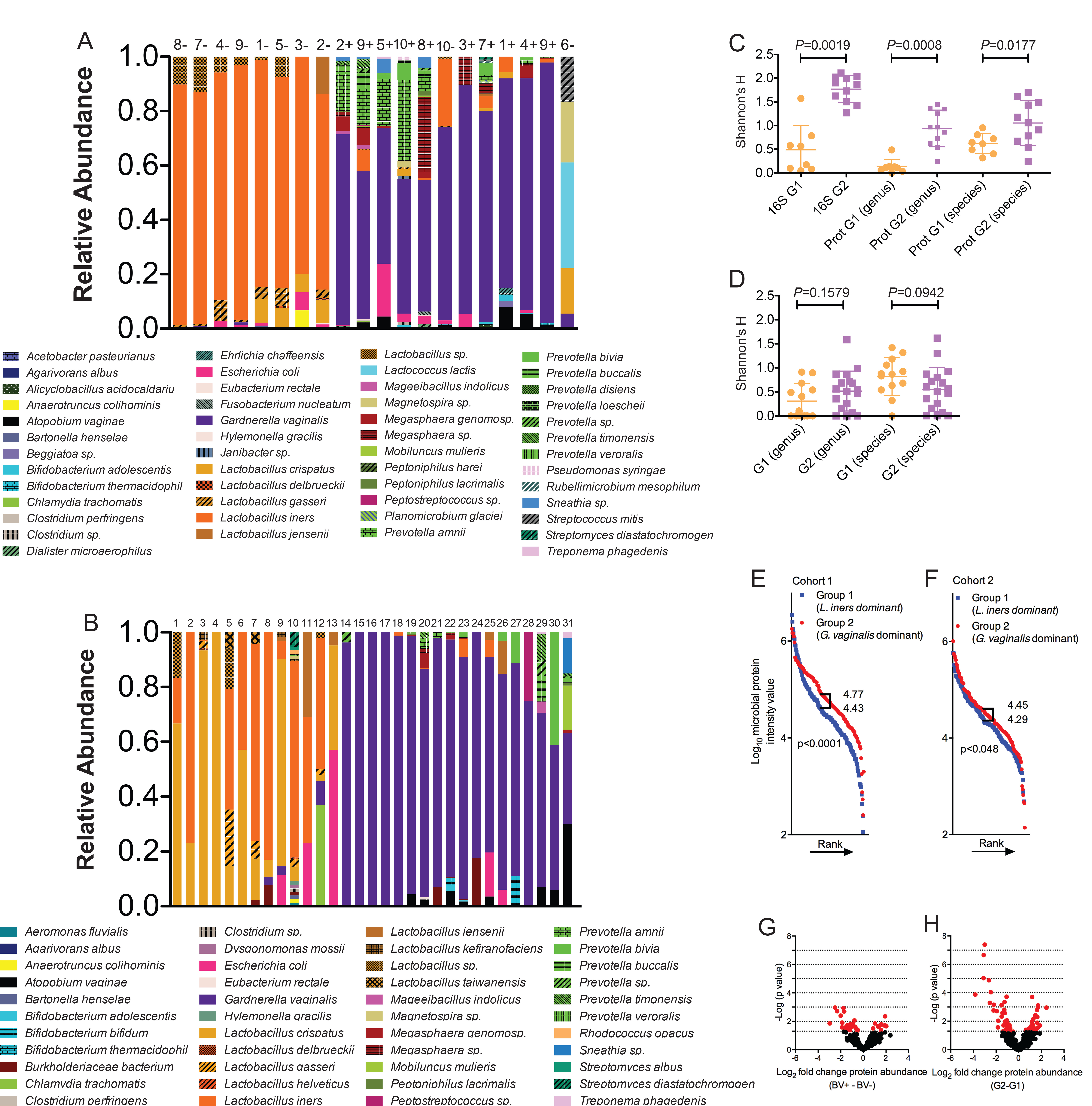

Supplement: S1 Fig — (A) Bacterial species and proteome diversity for Cohort 1. Most of the G1 communities were dominated by L. iners, while G. vaginalis, P. amnii, Megasphaera sp., and others dominated the G2 communities. (B) Bacterial species and proteome diversity for Cohort 2. G1 communities were dominated by either L. iners, L. crispatus, but also had the presence of L. gasseri, and L. jensenii. G2 communities were heavily dominated by G. vaginalis. (C) Shannon Diversity detected for Cohort 1. The G2 group always had higher diversity than the G1 group. (D) Shannon Diversity detected for Cohort 2. There was no difference in the diversity between the G1 and G2 groups. (E/F) Total bacterial proteome abudance differences between women with either a G. vaginalis or L. iners bacterial proteome profile for Cohort 1 (E) and Cohort 2 (F). These graphs show bacterial protein levels (y axis) as a function of abundance rank (x axis) in decreasing order. Median levels are shown (log10), and student t-tests (Cohort 1: paired t test, parametric; Cohort 2: unpaired t test, parametric) were used to determine statistical differences. G. vaginalis-dominant profiles always had increased protein load compared to those dominated by Lactobacillus. (G/H) Volcano plots depicting host protein expression differences using clinical BV status (G) or bacterial community profile (H). The Y-axis of the volcano plot denotes statistical significance, and the x-axis the fold-change (FC). Comparison of G1 and G2 community profiles, rather than BV criteria, yielded more statistically relevant differences between the host proteome. (TIF) [file ppat.1005889.s001.tif]

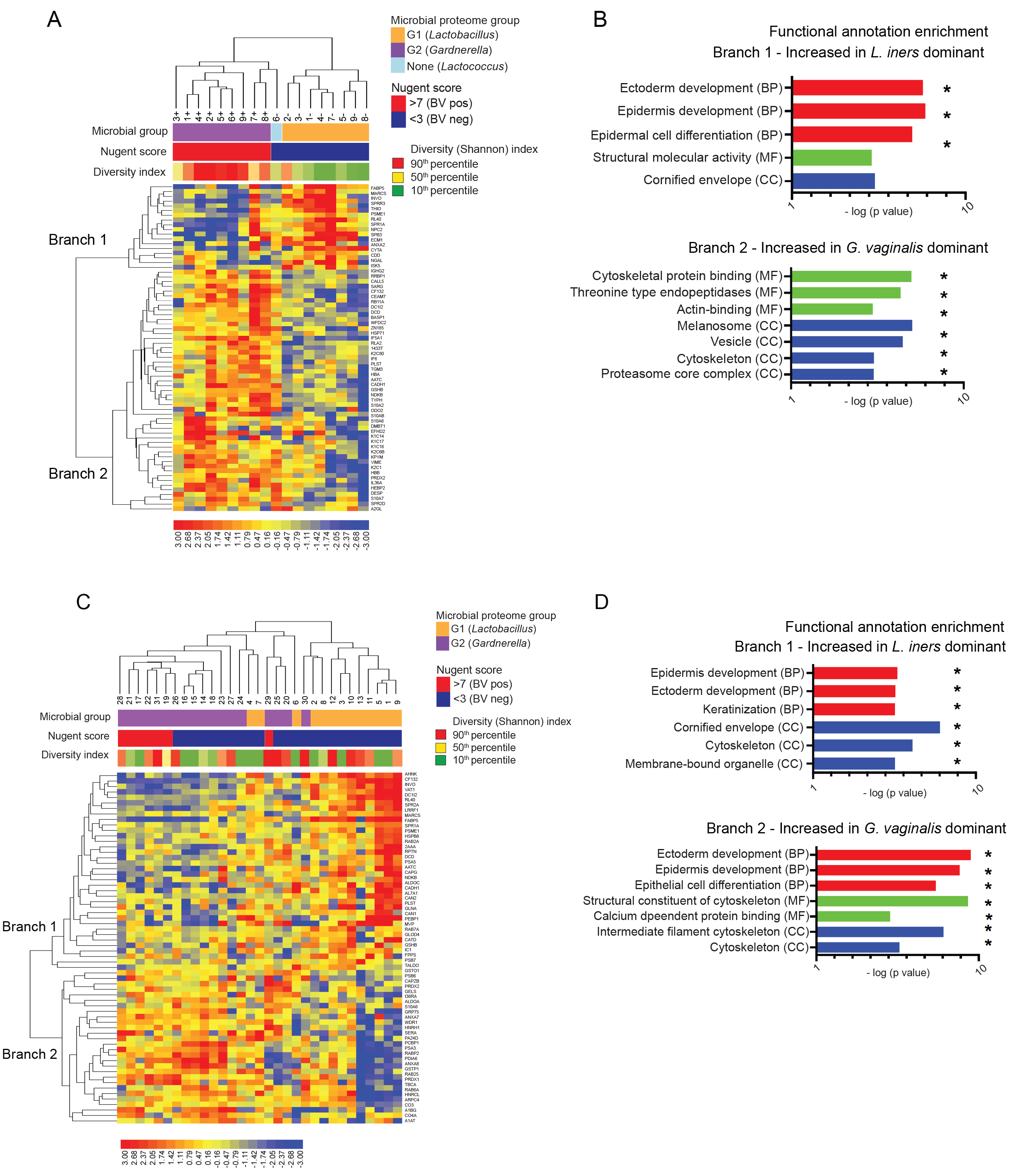

Supplement: S2 Fig — Heatmaps of differentially abundant host proteins (P<0.05) in cervicovaginal secretions of women based on vaginal bacterial communities. (A) Differentially abundant host proteins for Cohort 1; (B) Functional annotation of differentially expressed proteins based on their gene ontology for Cohort 1. (C) Heatmap of differentially abundant host proteins for Cohort 2; (D) Functional annotation of differentially expressed proteins based on their gene ontology for Cohort 2. BP: Biological process; CC: Cellular component; MF: Molecular function. (TIF) [file ppat.1005889.s002.tif]

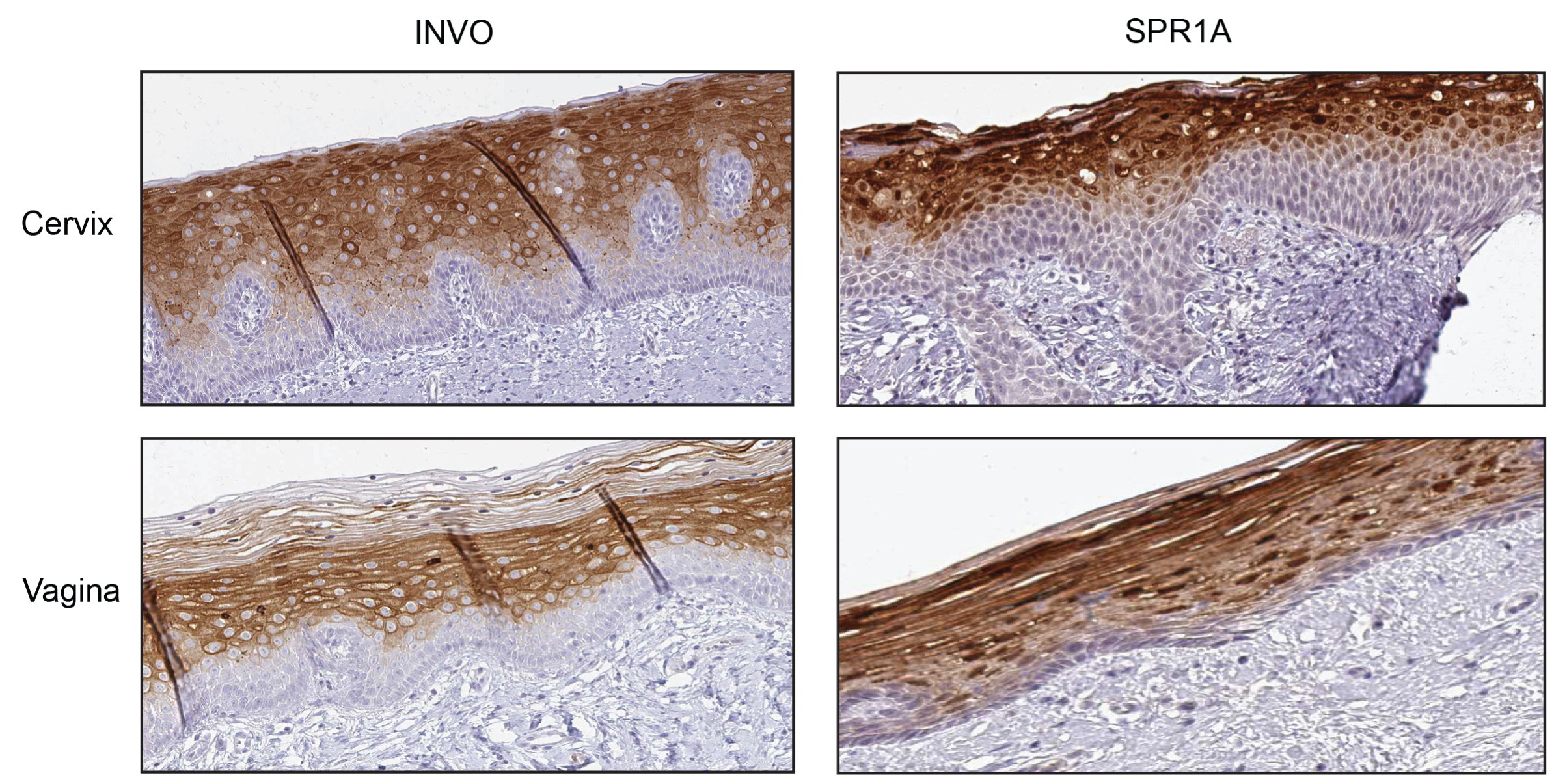

Supplement: S3 Fig — Immunohistochemical analysis of cervical and vaginal tissue revealed prominent expression of INVO and SPR1A in top layers of the squamous epithelium in cervix and vagina, with both showing association with the cytoplasm and nucleus membrane. Protein was not detected in glandular cells in cervix/endocervix. (TIF) [file ppat.1005889.s003.tif]
