# Supplementary material for: Microbiome Composition and Function Drives Wound-Healing Impairment in the Female Genital Tract
Source: PLoS Pathog. 2016 Sep 22;12(9):e1005889. doi: 10.1371/journal.ppat.1005889 (PMC5033340; doi:10.1371/journal.ppat.1005889)
Supplement: S1 Table — (DOCX) [file ppat.1005889.s006.docx]

**Supplemental Table S1. Amsel criteria for Cohort 2 (North America).**

| **Variable** | **G1** | **G2** | ***P*** |
| --- | --- | --- | --- |
| Vaginal pH (median) | 4.4 | 5.4 | 0.0005 |
| Positive Wiff test | 0/13 (0%) | 3/18 (16.7%) | 0.18 |
| Clue cells on wet mount | 0/13 (0%) | 10/18 (20.1%) | 0.001 |
| White blood cell present | 4/13 (30.7%) | 7/18 (36%) | 0.012 |
