# Supplementary material for: Microbiome Composition and Function Drives Wound-Healing Impairment in the Female Genital Tract
Source: PLoS Pathog. 2016 Sep 22;12(9):e1005889. doi: 10.1371/journal.ppat.1005889 (PMC5033340; doi:10.1371/journal.ppat.1005889)
Supplement: S2 Table — (DOCX) [file ppat.1005889.s007.docx]

**Supplemental Table S2. Proteins determined to be significantly differentially abundant between G1 and G2 in Cohort 1 (BH *P*: Bejamani-Hochberg corrected *P* value; *P*<0.001)**

| **Protein name** | **Species** | ***P*** | **BH *P*** |
| --- | --- | --- | --- |
| Glyceraldehyde-3-phosphate dehydrogenase | *L. iners* | 2.63E-08 | 7.85E-05 |
| Pyruvate kinase | *L. iners* | 8.06E-07 | 1.57E-04 |
| L-lactate dehydrogenase | *L. iners* | 1.43E-05 | 2.35E-04 |
| 60 kDa chaperonin (GroEL protein) | *L. iners* | 1.50E-05 | 3.14E-04 |
| Enolase | *L. iners* | 3.15E-05 | 3.92E-04 |
| Elongation factor Tu | *G. vaginalis* | 5.31E-05 | 4.71E-04 |
| Glucose-6-phosphate isomerase | *G. vaginalis* | 9.37E-05 | 6.28E-04 |
| Chaperone protein DnaK (Heat shock 70 kDa protein) | *L. iners* | 1.74E-04 | 7.06E-04 |
| MalE-type ABC sugar transport system periplasmic component | *G. vaginalis* | 3.08E-04 | 7.85E-04 |
| Fructose-1,6-bisphosphate aldolase | *L. iners* | 8.49E-04 | 8.63E-04 |
| Alpha-1,4 glucan phosphorylase | *G. vaginalis* | 9.10E-04 | 9.42E-04 |
