# Supplementary material for: Microbiome Composition and Function Drives Wound-Healing Impairment in the Female Genital Tract
Source: PLoS Pathog. 2016 Sep 22;12(9):e1005889. doi: 10.1371/journal.ppat.1005889 (PMC5033340; doi:10.1371/journal.ppat.1005889)
Supplement: S1 Methods — (DOCX) [file ppat.1005889.s008.docx]

Supplementary Appendix

This appendix has been provided by the authors to give readers additional information about their work.

Supplement to: Zevin A, *et al*. **Microbiome composition and function drives wound-healing impairment in the female genital tract**

**Supplemental Experimental Procedures:**

**Sample preparation for mass spectrometry**

Vaginal swabs were eluted with 2 x 250ul washes in PBS (pH 7.0) and the swab eluates (Cohort 1) or CVL samples (Cohort 2) were then centrifuged in SpinX tubes with a bonded fritted bottom (Corning, Corning, NY), and the protein content of these filtrates was then determined by BCA assay (Novagen, Bilerica, MA). 100µg of protein from each sample was then individually denatured with urea exchange buffer (8M Urea GE HealthCare, 50mM HEPES Sigma, pH 8.0) for 20 minutes at room temperature. These mixtures were then placed into 10 kDa cutoff Nanosep filter cartridges (Pall, Port Washington, NY), and centrifuged. Proteins were then reduced with 25mM dithiothreitol (Sigma) for 20 minutes, alkylated with 50mM iodoacetamide (Sigma) for 20 minutes at room temperature, and washed three times with urea exchange buffer and two times with 50mM HEPES buffer. Proteins were then digested by addition of 2 μg of Trypsin (Promega) and the samples were incubated at 37˚C overnight in the cartridge. Peptides were eluted from the filter with 50 mM HEPES, and dried using vacuum centrifugation. Reversed-phase liquid chromatography (high pH RP, Agilent 1200 series micro-flow pump, Water XBridge column) was employed to desalt and remove detergents from the peptides using a step-function gradient as described previously (Birse et al., 2015). Finally, the eluted peptide fractions were dried using vacuum centrifugation and stored at -80°C.

**DNA Extraction**

Total genomic DNA from eluted swab (Cohort 1) or CVL (Cohort 2) samples was extracted using the DNeasy Blood and Tissue Kit (Qiagen, Valencia, CA) with modifications to enhance lysis, as previously described (Zevin et al., 2016). Cell pellets were resuspended in 200 μL of lysis buffer (30 mM Tris·HCl, 10 mM EDTA, 200 mM sucrose, pH 8.2) and incubated the mixture at 65°C for 10 minutes. Chicken egg white lysozyme (Sigma Aldrich, St. Louis, MO) was then added to a final concentration 10 mg/mL and the samples were incubated for 1 hour at 37°C. Next, SDS was added to a final concentration of 1% (w/v) and samples were incubated at 56°C for 10 minutes. Finally, 25 μL proteinase K and 200 μL buffer AL (Qiagen) were added and the samples were incubated at 56°C for 30 minutes. After these additional lysis steps, DNA extraction was completed according to the manufacturer’s (Qiagen) instructions.

**16S rRNA gene sequence analysis**

Paired-end reads were combined using the PANDAseq assembler (Masella et al., 2012) and 1,444,291 reads with an average length of 257 base pairs were obtained. 16S rRNA gene sequence data was analyzed using the QIIME software package(Caporaso et al., 2010). Sequences were initially clustered into operational taxonomic units (OTUs) at 97% similarity and a representative sequence was selected for each OTU. Taxonomy was then assigned to the representative sequences by aligning sequences to the SILVA 111 release database (Pruesse et al., 2007) using the BLAST assignment method.

**Multivariate modeling**

K-fold cross-validation was used to determine the optimal value of the tuning parameter for Least Absolute Shrinkage and Selection Operator (LASSO) such that the resulting model had the lowest possible mean squared error for prediction. Partial Least-Squares Discriminant Analysis (PLSDA) was then used to determine how these LASSO-selected features classified our phenotypes of interest (G1 vs G2) (Eigenvector PLS toolbox, Eigenvector Research Inc.). Data were then normalized using mean centering and variance scaling prior to model construction. Cross validation was then performed by iteratively excluding random subsets (in groups equal to the number of LASSO selected features) during model calibration, and then used the excluded data to test model predictions. Cross-validation enabled us to determine if our LASSO-selected model was the best out of the 10,000 models tested.

**Preparation of bacteria and bacterial culture supernatants**

A standard curve relating optical density at 595nm (OD_595_) to total cell numbers using a spectrophotometer was initially generated. To prepare bacteria for the wound-healing assay, overnight cultures of *L. iners* and *G. vaginalis* were grown in NYCIII medium. For experiments requiring live bacteria, 1 mL of the overnight cultures was centrifuged at 13,000 RCF for 3 minutes, and the supernatant was removed and stored. Cell pellets were then washed three times in 1 mL of 1X phosphate buffered saline (PBS) (GE Healthcare Life Sciences). To prepare culture supernatants, the initial OD_595_ of the overnight cultures were measured and the culture supernatants were diluted with fresh NYCIII medium such that the final solution obtained was representative of supernatant generated by 10^9^ bacterial cells/mL. The final supernatant solutions were then filtered through a pre-rinsed syringe filter with nominal pore size of 0.2µm (Pall).

**Wound-healing assay**

Wells of a 24-well tissue culture plate (Corning) were seeded with 50,000 HeLa cells in a volume of 500 µL DMEM supplemented with 10% (v/v) FBS, and 1% (v/v) pen/strep/amph B solution, and incubated at 37˚C under 5% CO_2_ for 24 hours or until a confluent cell monolayer had formed. The monolayers in each well were then scratched using a sterile P200 pipette tip and washed three times with 500µL of 1X PBS. To each well, 500 µL of DMEM supplemented with 10% (v/v) FBS was then added. To test the effects of bacterial culture supernatants, 100 µL of the diluted and filtered supernatants described above was added to the appropriate wells. For controls, 100 µL of fresh NYCIII medium was added to the appropriate wells.
